# Unearthing Antibiotic Resistance Associated with Disturbance-Induced Permafrost Thaw in Interior Alaska

**DOI:** 10.3390/microorganisms9010116

**Published:** 2021-01-06

**Authors:** Tracie J. Haan, Devin M. Drown

**Affiliations:** 1 Department of Biology and Wildlife, University of Alaska Fairbanks, Fairbanks, AK 99775, USA; tjhaan@alaska.edu; 2 Institute of Arctic Biology, University of Alaska Fairbanks, Fairbanks, AK 99775, USA

**Keywords:** antibiotic resistance, environmental resistome, RefSoil+, soil disturbance, permafrost thaw

## Abstract

Monitoring antibiotic resistance genes (ARGs) across ecological niches is critical for assessing the impacts distinct microbial communities have on the global spread of resistance. In permafrost-associated soils, climate and human driven disturbances augment near-surface thaw shifting the predominant bacteria that shape the resistome in overlying active layer soils. This thaw is of concern in Alaska, because 85% of land is underlain by permafrost, making soils especially vulnerable to disturbances. The goal of this study is to assess how soil disturbance, and the subsequent shift in community composition, will affect the types, abundance, and mobility of ARGs that compose the active layer resistome. We address this goal through the following aims: (1) assess resistance phenotypes through antibiotic susceptibility testing, and (2) analyze types, abundance, and mobility of ARGs through whole genome analyses of bacteria isolated from a disturbance-induced thaw gradient in Interior Alaska. We found a high proportion of isolates resistant to at least one of the antibiotics tested with the highest prevalence of resistance to ampicillin. The abundance of ARGs and proportion of resistant isolates increased with disturbance; however, the number of ARGs per isolate was explained more by phylogeny than isolation site. When compared to a global database of soil bacteria, RefSoil+, our isolates from the same genera had distinct ARGs with a higher proportion on plasmids. These results emphasize the hypothesis that both phylogeny and ecology shape the resistome and suggest that a shift in community composition as a result of disturbance-induced thaw will be reflected in the predominant ARGs comprising the active layer resistome.

## 1. Introduction

The rapid evolution and spread of antibiotic resistance is one of the greatest challenges faced in public health today. Antibiotic resistance impedes the successful treatment of bacterial infections by reducing antibiotic efficacy, increasing disease burden, mortality rates, hospitalization time and cost [1]. On an evolutionary time scale, the extensive prevalence of resistant phenotypes in human pathogens is a recent event, driven by the large-scale production and widespread use of antibiotics in clinical, agricultural, and veterinary settings [2,3]. Even when antibiotic stewardship is instilled (i.e., antibiotic use is confined to essential needs), antibiotics, or pollutants such as heavy metals that coselect for resistance [4], are dispersed within microbial habitats, thereby generating selective pressures that increase the abundance of resistant strains and their associated antibiotic resistance genes (ARGs). It was originally thought that the genetic variability driving resistance was primarily caused by mutational modification to antibiotic targets, and thus, would remain clonal [5]. However, it is now evident that mutational-driven resistance is a weaker force compared to ARGs acquired via horizontal gene transfer (HGT) [6]. In pathogens, resistance genes can be acquired from diverse microbial habitats and taxa [5,7], including bacteria from pristine environments free of antibiotics introduced via human activities [8]. It is therefore important to assess which bacterial taxa and microbial biospheres are the predominant contributors to the evolution of resistance in pathogens [9]. 

Soils, one of the most diverse microbial habitats on earth, are a vast repository of both antibiotic-producing and coevolved resistant microbial taxa. Antibiotic production is thought to have originated in soils from 2 Gyr to 40 Myr ago, suggesting that resistance has undergone concomitant evolution over a similar timeframe [10,11]. The evolutionary origin of resistance is also supported by studies that have unveiled soils unpolluted by human activity that harbor diverse resistance mechanisms to modern antibiotics, such as 30,000-year-old Beringian permafrost sediments [8]. Moreover, bacteria such as *Streptomyces*, an Actinomycete genus that produces around two-thirds of clinically used antibiotics, are abundant in soils [12,13]. The presence of these antibiotic-producing genera is thought to promote the evolution, and potential dissemination via HGT, of clinically relevant resistance genes from soils. Recent studies have reported ARGs in soil-borne bacteria identical to those circulating in pathogens, suggesting that HGT has occurred [14,15]. These shared ARGs between soil-borne and pathogenic bacteria emphasize a potential role soils have in the evolution and dissemination of resistance. However, to assess the risks posed by soil-borne ARGs, more attention should be paid to their distribution globally, which environments favor growth of bacteria harboring ARGs, and conditions that promote mobility of ARGs such as plasmid carriage [16]. By examining soils affected by environmental change, we can provide insights into both the global distribution and how change affects resistance determinants that may emerge in pathogens.

Alaskan soils are one environment undergoing unprecedented change, as warming within the arctic is occurring 2.5 times faster than in the rest of the globe [17]. This warming has triggered a rise in the frequency of soil disturbance events, such as wildfires and thermokarst formation, which are of particular concern for Alaska, since approximately 85% of land is underlain by discontinuous permafrost [18,19,20]. With both climatic and human-driven soil disturbances, near-surface permafrost thaw is augmented, shifting the physical and chemical properties of the overlying active layer soils [21]. Alterations to pH, moisture, and nutrients in the active layer impact ecosystem functions while shifting microbial community composition [22]. This shift in both biotic and abiotic factors has the potential to enrich ARGs by selecting for their host taxa. For example, the phylum Proteobacteria is significantly enriched with plasmid-borne resistance determinants compared to other bacterial phyla [23] and over-represented in terms of abundance in active layer soils associated with disturbance-induced thaw [24]. This connection between ARGs, phylogeny, and community shifts observed in disturbed soils of Alaska makes it imperative to assess how thaw will affect bacteria from active layer soils as a reservoir of resistance.

The Fairbanks Permafrost Experiment Station (FPES) is a long-term research site established by the Army Corps of Engineers in 1945 containing ice-rich permafrost typical of Alaska [21]. FPES has three levels of increasing soil disturbance with minimal thaw in the undisturbed site and up to 9.8 m in the most-disturbed site. The location’s distinctive gradient of disturbance-induced thaw makes it fitting for research on the effect of disturbance on permafrost, vegetation, soils, and microbial communities. Previous culture-independent metagenomic analyses conducted at this site found distinct shifts in microbial community composition, such as an enrichment of Proteobacteria in the disturbed soils [24], necessitating more research into how this site has affected antibiotic resistance.

The goal of this study is to assess how soil disturbance, and the subsequent shift in community composition, will affect the types, abundance, and mobility of ARGs that compose the Alaskan active layer resistome. To explore the effects of both phylogeny and ecology on ARGs from active layer soils of Alaska, we identified ARGs in the whole genome sequences of bacteria cultured from FPES active layer soils. We then put these FPES isolates into a global context by comparing ARGs to those identified in bacteria from a database of global soil bacteria, RefSoil+ [25]. This larger database containing both the whole genomes and plasmid sequences of bacteria cultured from global soil habitats makes it possible to investigate distinguishing features of ARG ecology in Alaskan isolates, such as the plasmid carriage and ARG abundance by taxa. Overall these analyses will allow us to gain insights into the role both biotic and abiotic factors associated with disturbance-induced permafrost thaw will have on antibiotic resistance. By cataloguing ARGs and their host taxa, this work also contributes to the critical knowledge gap regarding the global distribution of ARGs from the environmental biosphere that have the potential to spread to clinically significant bacteria and compromise health.

## 2. Materials and Methods

### 2.1. Permafrost Thaw Gradient

**Figure 1:**
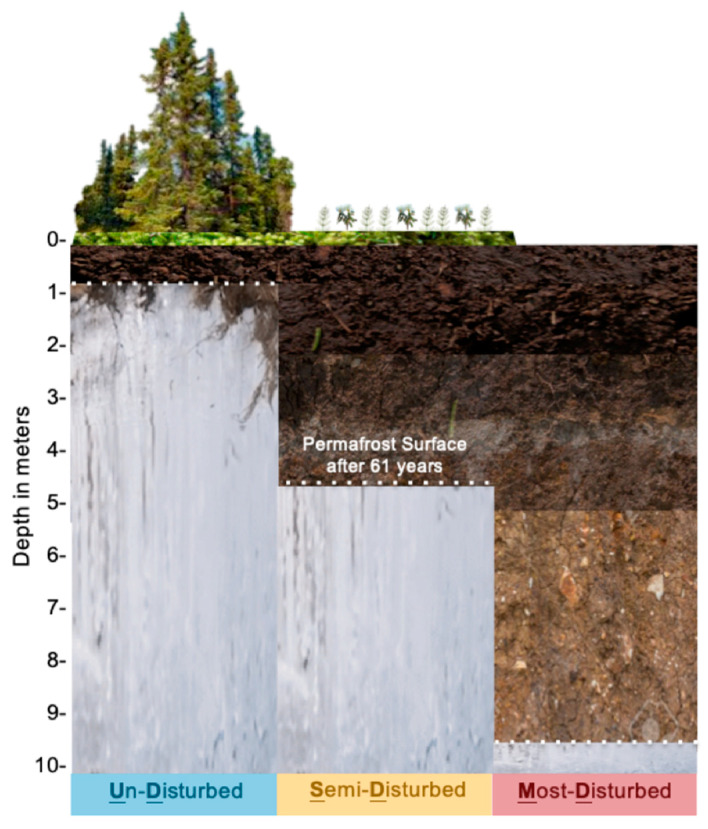
Diagram of the disturbance level sites (Undisturbed = UD, Semi-Disturbed = SD, Most-Disturbed = MD) at the Fairbanks Permafrost Experiment Station (FPES) and the subsequent depth of permafrost thaw after 61years.

The Fairbanks Permafrost Experiment Station (FPES) is an ice-rich permafrost site in Interior Alaska (64.875646° N, 147.668981° W), established by the Army Corps of Engineers as part of the Cold Regions Research and Engineering Lab. The site consists of three 3721 m^2^ Linell plots [26] with increasing levels of disturbance, now known as the Un-Disturbed (UD), Semi-Disturbed (SD), and Most-Disturbed (MD) sites (Figure 1). In 1946, the three sites were established to simulate soil disturbance events, such as wildfire or anthropogenic disturbance, on permafrost degradation by clearing of vegetation. The UD site was left undisturbed to preserve the subarctic taiga forest, whereas the SD site had the surface vegetation cleared while roots and soil organic matter were left intact and the MD site had both surface vegetation and organic matter removed. In 2007 the UD site, which is now monitored as part of the Circumpolar Active Layer Monitoring Network (CALM), was found to have little to no thaw since 1946 whereas the SD and MD had up to 4.7m and 9.8m respectively [21]. These results suggested that permafrost degradation is dependent on both time and surface vegetation which has major implications when it comes to increasing frequency of disturbance events, like wildfires and thermokarst formation, influenced by climate change.

Vegetation at the FPES is typical of the Alaskan Interior-subarctic taiga forest. The undisturbed site is a relatively open black spruce stand (*Picea mariana*) with an understory of continuous thick moss layer interspersed with low-bush cranberry (*Vaccinium vitis-idaea*) and Labrador tea (*Rhododendron groenlandicum*). The UD site can be classified as mesic with a soil organic layer thickness ranging 2 to 35 cm thick, with little to no thaw during maximal permafrost thaw [27]. The semidisturbed site is now a mix stand dominated by black spruce, Alaskan paper birch (*Betula neoalaskana*), and willow (*Salix alaxensis*). The understory contains a mixture of Labrador tea (*Rhododendron groenlandicum*), Peltigera lichen, roses, horsetail, cloudberry, and small amounts of grass with little litter cover. The MD site is an open shrub land dominated by willows (*Salix alaxensis*) and a developing over story up to 5 m tall of Alaskan birch and black spruce (*Picea mariana*). The understory contains many grasses, clovers, horse-tail (*Equisetum*), and some bare ground. There is no permafrost within the top 4.7 m in either disturbed sites [21].

### 2.2. Bacterial Culturing

In the September of 2017, we collected two 10-cm wide by 20-cm deep soil cores from each FPES site with a sterilized soil probe. Prior to coring, the top layer of moss and vegetation were removed. Soil cores were then extracted and immediately stored in a cooler throughout sample collection. Soil cores were moved to a +4 °C fridge until processing 24 h later. To prevent contamination from exogenous cells on the exterior of the soil core, the outer portion of each was removed using a sterile scalpel. The interiors of each core were then sub-sampled using sterile forceps along a depth gradient at intervals of about 2.5 cm for 20 cm to generate a total of 1 g of soil. The 1 g of soil was used to inoculate 100 mL tryptic soy broth (TSB) to produce an enrichment culture. After 48 h at 22 °C, we plated serial dilutions (1:10, 1:100, 1:1000) of the enrichment culture three times for each sample and incubated the plates at three temperatures (+4 °C, +12 °C, and +20 °C), for a total of twelve plates per sample, until distinct colony formation was observed. Ten discrete colonies were chosen at random from each temperature and FPES site by using a random number generator to count colonies across transects moving across the plate horizontally from the top of the plate to the bottom. Only discrete colonies (i.e., colonies without overlapping colony growth) were counted along the transect lines. This process yielded 90 total colonies, 30 per FPES site. Each colony was isolated and purified using three rounds of streak plate method.

### 2.3. Antibiotic Susceptibility Testing

We screened each isolate for antibiotic resistance using the Kirby-Bauer disk diffusion method [28]. In brief, this method uses paper disks with a fixed concentration of antibiotic that diffuses into agar generating a region where susceptible bacteria cannot grow, called the zone of inhibition. The diameter of this circular zone is then measured and compared to breakpoints established for clinical isolates with values based on isolate taxonomy. In this study, we used five antibiotics, i.e., tetracycline, erythromycin, kanamycin, chloramphenicol, and ampicillin, with each representing a distinct antibiotic class. Standards for antibiotic disks and associated breakpoints were used from the US Clinical and Laboratory Standards Institute M100, 30th ed. Breakpoints established for *Enterobacterales* were used for *Serratia*, *Pantoea*, and *Erwinia* isolates, *Pseudomonas* spp. breakpoints for *Pseudomonas* isolates, and *Enterococcus* spp. breakpoints for *Bacillus* and *Exinguobacterium* isolates to determine if an isolate was susceptible, intermediate, or resistant to each antibiotic tested (Table A1). Isolates without breakpoints to specific antibiotics due to physiological characteristics that render that genus intrinsically resistant, such as in the case of *Pseudomonas* and ampicillin, were removed from subsequent analysis. 

### 2.4. Whole Genome Sequencing, Assembly, and Taxonomic Classification

From each purified isolate, we inoculated a liquid culture of TSB, incubated it at 22 °C overnight, and used 1.8 mL of this liquid culture to extract genomic DNA using the DNeasy UltraClean microbial kit (Qiagen, Venlo, The Netherlands) following manufacture protocols. This DNA was then used for sequencing on both Illumina and Oxford Nanopore Technology [ONT] platforms. For Illumina sequencing, we used a Nextera XT library (Illumina, San Diego, CA, USA) prepared by the Genomics Core Lab at the University of Alaska Fairbanks to sequence on an Illumina MiSeq platform with version 3 reagents. We trimmed adapters from Illumina reads with TrimGalore version 0.5.0 [29]. For ONT long reads, a combination of SQK-RBK004 and VSK-VSK002 library preparation (ONT) was employed (Table S1). These libraries were then sequenced on a MinION device (ONT) with r9.4.1 flow cells (FLO-MIN106) for 48–72 h. We base called the raw data using Guppy v3.4.5 (ONT) specifying the high-accuracy model (-c dna_r9.4.1_450bps_hac.cfg) and default parameters. We de-multiplexed isolate samples using the guppy_barcoder function of Guppy with parameters to discard sequences with middle adapters (–detect_mid_strand_barcodes) and trim barcodes (–trim_barcodes). We used Filtlong v0.2.0 [30] to filter by length (≥50 bp; –min_length 50) and quality (Q) score (≥10; –min_mean_q 90). Flye version 2.7 [31] was used to assemble quality controlled ONT reads specifying nanopore raw reads (–nano-raw) and genome size of 5 mb (–genome-size 5m). The unicycler_polish tool of Unicycler version 0.4.8 [32] was used to polish the flye assemblies with the Illumina reads as input. For isolates TH26, TH81 and TH88, ONT long-read assemblies that were previously published in Haan et al. 2019 and Humphrey et al. 2019 respectively were used as inputs for unicycler_polish [33,34]. 

These assemblies were then annotated with RAST tool kit (RASTtk) in PATRIC v3.6.3 using the Genome Annotation Service [35]. 16S rRNA gene copies for each assembly were aligned using MAFFT v7.450 and consensus sequences were run through blastn version 2.10.0 against the NCBI 16S rRNA database. If the top five hits ranked by bit score were from the same genus, then taxonomy was assigned to an isolate at a genus level (Table 1).

### 2.5. Antibiotic Resistance Gene Identification

In this study we identified ARGs by annotating each whole genome assembly with the Comprehensive Antibiotic Resistance Database (CARD) version 3.0.9 using command line tool Resistance Gene Identifier (RGI) version 5.1.0 specifying input type contig (–t contig) with default parameters for BLAST alignment (–a BLAST) and strict and perfect hits only. In order to detect previously unknown homologs using detection models with curated similarity cut-offs while still ensuring the detected variant is a functional resistance gene rather than spurious partial hits, we used the strict algorithm rather than the algorithms that would select for exclusively perfect or loose hits [36]. Results from RGI were further quality controlled by removing any Antibiotic Resistance Ontology (ARO) hits defined as mutations or ARO hits with less than 50% coverage of the reference sequence unless cutoff on the edge of a contig. To determine if a hit was located on a chromosome or plasmid, contigs containing hits were run through blastn [37] in Geneious Prime version 2019.2.1 against bacteria (taxid = 2) from the RefSeq database. The top hit ranked by bit score was then used to determine if the contig was most similar to a known plasmid or chromosome sequence. 

### 2.6. RefSoil+ Comparison

RefSoil+ [25] genomes and plasmids were downloaded from NCBI using accession numbers available on the RefSoil+ github page [38]. Each RefSoil+ sequence was then run through RGI following the same protocol as the FPES analysis of ARGs. Genomes belonging to the matching genera (*Bacillus*, *Erwinia*, *Exinguobacterium*, *Pantoea*, *Pseudomonas*, and *Serratia)* as FPES isolates were used for comparison of the RefSoil+ and FPES resistance genes. The genus *Pseudomonas* contains the largest and most diverse species with eight distinct phylogenomic groups, because the species *Pseudomonas aeruginosa* is distinctive from other groups within our samples *aeruginosa* genomes were removed from FPES versus RefSoil+ analysis.

### 2.7. Data Analyses and Statistics

Tabular outputs from RGI were used to conduct statistical analyses in R Studio version 3.5.7 [39] and visualizations were generated with the R package ggplot2 version 3.2.1 [40]. In order to determine if ARGs were influenced by phylogeny or ecology of FPES sites, we examined statistical differences in the number of ARGs per genome between phyla and across thaw and then compared FPES isolates to correspondent genera from RefSoil+. Kruskal-Wallis one-way analysis of variance was used to test significance of FPES site and phylum as predictors and then a post hoc test using nonparametric Wilcoxon test was used to test for significant differences between groups. We used Akaike Information Criterion model selection to see which Poisson distributed generalized linear model with phylum, FPES site, and both as predictors of the number of resistance genes per isolate has the best-fit. We used a heatmap generated with the R package pheatmap version 1.0.12 [41] to visualize the distribution of ARG hits across phyla and FPES sites. To generate the heatmap, we normalized ARG counts within each group that consisted of phylum and FPES site (e.g., Proteobacteria from MD) by the number of isolates in that group, and then scaled each normalized count for each resistance gene to generate z-scores. Dendograms grouping each column and row were based on Pearson correlation.

## 3. Results

### 3.1. Assessment of Antibiotic Susceptibility in FPES Isolates

**Figure 2:**
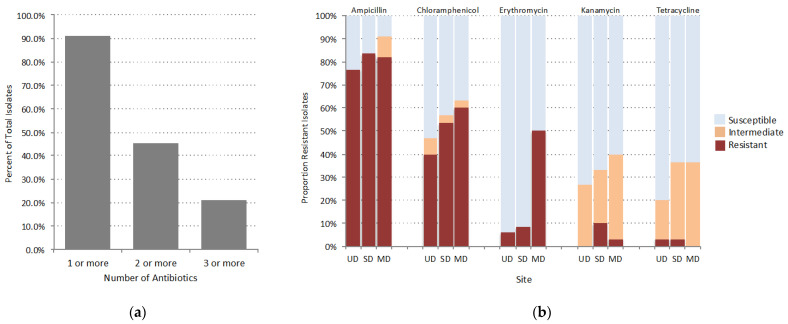
(**a**) Percent of total isolates with at least intermediate resistance to one or more, two or more, and three or more of the antibiotics tested. (**b**) Proportion of isolates at each FPES thaw site with the associated level of susceptibility (susceptible = light blue, intermediate = orange, resistant = red) to each antibiotic tested based on CLSI breakpoints.

Widespread resistance was observed in the isolates with 91.1% of the 90 total isolates exhibiting at least intermediate resistance to one of the five antibiotics tested, and 45.6% displaying at least intermediate resistance to two or more antibiotics (Figure 2a). Ampicillin had the highest prevalence of resistance (82.5%), followed by chloramphenicol (51.1%) and erythromycin (17.5%). Tetracycline had the lowest prevalence of resistance (2.2%). We observed a positive trend in the number of resistant isolates with disturbance-induced thaw for ampicillin, chloramphenicol, and erythromycin that was also observed for intermediate resistance to kanamycin and tetracycline (Figure 2b).

### 3.2. Genome Assembly Statistics 

The assembled genomes had a high mean percent completeness (98.91% ± 0.50), low percent contamination (1.16% ± 0.26), and high N50 value (N50 = 3,639,582 ± 279,870 bp, N50_n_ = 13 ± 5 contigs) for the mean total length of assemblies (6,096,842 ± 946,004 bp). These results, outlined in Table S2, suggest high quality assemblies were produced.

### 3.3. Antibiotic Resistance Genes Identified in FPES Isolates

Across all FPES genomes RGI identified 379 significant hits comprising 27 CARD-based AROs (Table S3). Of these 379 hits, 30 had 100% sequence identity to CARD AROs and another 32 were highly similar (sequence identity > 90%). Four genes hits had full length coverage of the reference sequence along with 100% sequence identity, two encoding aminoglycoside inactivating enzymes *AAC(6’)-32* and *AAC(6’)-Ir* and two encoding *bcrC*, an undecaprenyl pyrophosphate related protein. However, overall mean percent identity across all hits identified was 68.4% ± 19.

Genes encoding proteins for antibiotic efflux, antibiotic inactivation, antibiotic target modification, and antibiotic target protection were observed. The top two most abundant resistance genes identified across isolates were genes encoding proteins for antibiotic efflux. The most abundant efflux pump was *adeF*, a resistance-nodulation-cell division efflux pump that confers multi-drug resistance. There were multiple copies of this gene present in Proteobacteria with 176 chromosomally encoded gene copies distributed across the 100% of Proteobacteria isolates with a high coverage of the gene across assemblies (% length of reference sequence = 99.02 ± 2.902) and variable percent identity (% identity = 52.283 ± 11.822). The second most abundant resistance gene was *AbaQ* (% identity = 72.7 ± 0.427% length of reference sequence = 101.36 ± 0.109), a gene encoding the major facilitator superfamily efflux pump associated with the extrusion of quinolone-type drugs in *Acinetobacter baumannii. AbaQ* was observed across all FPES sites in 78.4% of *Pseudomonas* isolates.

After antibiotic efflux, genes encoding antibiotic inactivating enzymes were the most abundant. These genes were found in isolates from across all FPES sites and genera sampled except *Exinguobacterium*. *FosB* (% identity = 89.055 ± 2.281%, length of reference sequence = 105.012 ± 6.992) was the most abundant gene encoding an antibiotic inactivating enzyme with 25 chromosomally encoded gene copies present in 83% of *Bacillus* isolates across all thaw sites. We also observed beta-lactam inactivating genes from four distinct beta-lactamase families (Table S1). The Bc beta-lactamase gene family had the highest abundance (n = 20) all encoding *BcII,* a zinc metallo-beta-lactamase that hydrolyzes a large number of penicillins and cephalosporins. *BcII* gene copies in our samples were confined to the genus *Bacillus* and found to be both highly similar (% identity = 90.755 ± 0.567) with high gene coverage (% length of reference sequence = 100.39 ± 0) to *BcII* homologs in the CARD database. 

Genes encoding target alteration were the least abundant mechanism of resistance (n = 25) and included the genes *armA*, *bcrC*, *MCR*-*4.1*, *gyrB*, *PmrF*, *sgm*, and *vanJ* which are associated with resistance to aminoglycosides, peptide, glycopeptide, and fluoroquinolone antibiotics. A notable target alteration gene found was the mobilized colistin resistance (MCR) phosphoethanolamine transferase. MCR is a gene superfamily tracked by the Center for Disease Control and Prevention that confers resistance to the last resort antibiotic colistin, a critical antibiotic for treating carbapenem-resistant *Enterobacteriaceae*. We found two significant gene hits for *MCR-4.1*, however they were fragmented on the edge of contigs and thus had low coverage (% length of reference sequence = 10.63 ± 1.174) but 100% sequence identity.

### 3.4. Influence of Phylogeny and Disturbance-Induced Thaw on ARGs

**Figure 3:**
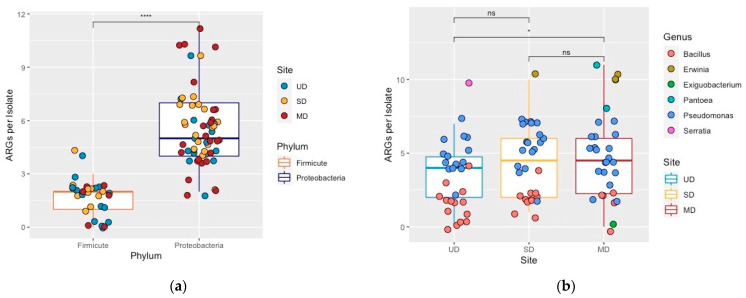
(**a**) Boxplot displaying the number of antibiotic resistance genes per isolate by phylum (Kruskal-Wallis *p* = 2.8 × 10^−13^) with points representing an isolate color-coded by FPES site. (**b**) Boxplot displaying the number of antibiotic resistance genes per isolate by FPES site ((Kruskal-Wallis *p* = 0.083)) with points representing an isolate color-coded by phylum. Wilcoxon test between group significance *p* < 0.0001 ****, *p* < 0.05 *, ns >0.1.

Although the abundance of ARGs increased with FPES disturbance levels (UD = 101, SD = 133, MD = 145), we found the number of CARD hits per isolate was highly significant by phylum (Kruskal-Wallis *p* = 2.8 × 10^−13^; Figure 3a) rather than FPES site (Kruskal-Wallis *p* = 0.083; Figure 3b). Although FPES site was not significant as a predictor, there was a significant difference between the numbers of ARGs per isolate from the undisturbed to most-disturbed sites (Wilcoxon *p* = 0.045). When comparing generalized linear models containing FPES site, phylum, and both site and phylum as predictors of ARGs per isolate we found that the model with phylum alone had the best fit (AIC = 349 phylum; 429 FPES site; 351 both).

**Figure 4:**
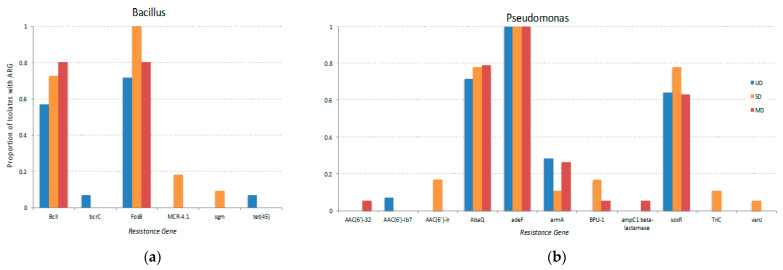
The proportion of (**a**) *Bacillus* and (**b**) *Pseudomonas* isolates from each FPES site that contain each ARG.

**Figure 5:**
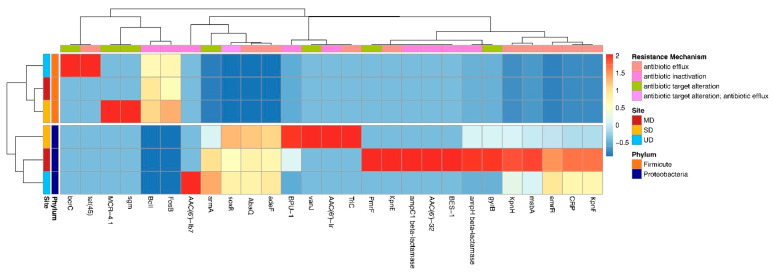
Heat map displaying the z-score by column of ARG count normalized by number of isolates in each group (phylum and FPES site). Annotation colors on the side show FPES site and phylum of each group and annotations on top show the resistance mechanism of the associated ARG. Dendograms display clustering based on Pearson correlation.

When examining the types of genes more in depth by phylogeny, there is a distinct set of ARGs found within each genus such as *FosB*, *BcII* in *Bacillus* (Figure 4a) and *adeF*, *armA*, and *soxR* in *Pseudomonas* (Figure 4b). Overall *Bacillus* isolates’ core resistance genes were comprised of primarily antibiotic inactivation genes, *Pseudomonas* was antibiotic efflux and target alteration, and *Erwinia* contained antibiotic efflux, target alteration, and inactivation. These sets of core ARGs present in some taxa and absent in the others are what appeared to cause ARGs to cluster more strongly by phylum rather than thaw site in the heatmap (Figure 5). Although there was ARGs found across FPES sites in multiple genera (Table S4), some genes were observed exclusively in one taxon and site, such as MCR-4.1 in *Bacillus* from the semidisturbed site (Figure 4a). 

### 3.5. Comparison of ARGs in RefSoil+ and FPES Genomes from Corresponding Genera

**Figure 6:**
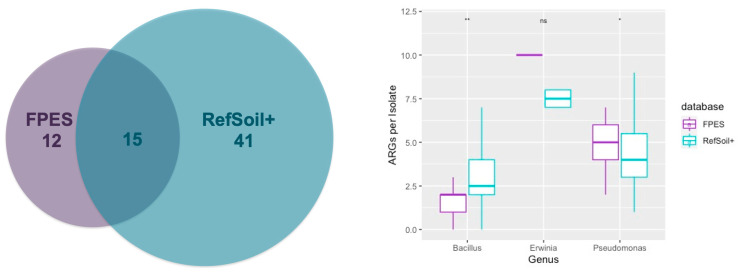
(**a**) Types of ARGs by database (**b**) Boxplot of the number of antibiotic resistance genes per isolate by genus and database (FPES vs RefSoil+). Wilcoxon test significance *p* < 0.01 **, *p* < 0.05 *, >0.1 ns.

Across the equivalent genera, RefSoil+ and FPES had 15 similar ARGs variants, 41 unique to RefSoil+ and 12 unique to FPES (Figure 6a). The similar ARGs were primarily genes determined to be the more abundant ARGs in FPES isolates whereas the distinct genes were often rare variants (i.e., only one copy across all isolates). When comparing by genus and database we found there were significant differences in number of ARGs per isolate between *Pseudomonas* and *Bacillus* isolates, which was higher in FPES for *Pseudomonas* and higher in RefSoil+ for *Bacillus* (Figure 6b). Across the 90 FPES isolates, 4 ARG variants with 6 gene copies were identified in plasmid sequences whereas there was no significant plasmid hits across all 127 RefSoil+ isolates examined from corresponding genera. The plasmid-borne ARGs from FPES included a BES-1 beta lactamase, two antibiotic efflux pumps (*TriC* and *KpnF*), and an undecaprenyl pyrophosphate related protein (*bcrC*) (Table 2). 

## 4. Discussion

The Alaskan soil bacteria in this study harbored a diverse array of resistance determinants from all major mechanisms of antibiotic resistance, corroborating findings that suggest ARGs are ancient in origin and ubiquitous in soil-dwelling bacterial taxa [8,42]. Although we cannot draw direct functional conclusions from genomic data, such as if a resistance gene will be transcribed in an isolate, we did find that the high abundance of beta-lactam resistance genes in isolates directly corresponds with the high proportion of phenotypic resistance to the beta-lactam antibiotic screened, ampicillin. Of the seven isolates susceptible to ampicillin only two had a hit for a beta-lactamase gene, whereas the prevalence in the resistant isolates was much higher with 24 of the 32 resistant isolates encoding a beta-lactamase gene (Table S4). In terms of the effect of disturbance-induced thaw associated with FPES sites, we observed a positive trend in both the proportion of resistant isolates (Figure 2) and abundance of ARGs with disturbance level. There were also significantly more ARG copies per isolate in the MD site compared to the UD (Figure 3a). However, this difference is likely a result of the increasing number of randomly sampled Proteobacteria (including *Erwinia*, *Pseudomonas*, *Pantoea*, and *Serratia*) with thaw (n = 15 UD; 19 SD; 24 MD) since isolates from the Proteobacteria phyla had a significantly higher number of ARGs per genome compared to the Firmicutes sampled (*Bacillus* and *Exiguobacterium*) (Figure 3a).

Based on AIC model selection, we found that phylum had a stronger effect on the number of ARGs per isolate than FPES site or both FPES site and phylum. The link between host phylogeny and ARG abundance demonstrates how a loss of Firmicutes and enrichment of Proteobacteria as a result of community shifts could increase the abundance of ARGs within a community. Although we cannot say from this specific dataset of cultured isolates which taxa are enriched across FPES sites, previous analyses conducted on uncultured metagenomic data from across 48 FPES cores in Seitz et al., 2020 identified over-representation of the phylum Proteobacteria in the disturbed cores and of the order *Bacillales* in undisturbed cores [24]. This enrichment in the metagenomic data parallels the enrichment of these taxa in this cultured subset of the community.

Along with the observed association of host taxa and ARG abundance, we found that the types of ARGs clustered by bacterial phylum rather than FPES site (Figure 4). Moreover, the predominant mechanisms of resistance (e.g., efflux, inactivation, target protection, and target alteration) were dependent on host taxa. This connection between host taxa and types of resistance determinants means that as microbial community composition shifts in response to permafrost thaw, so can the predominant taxa shaping the types and of ARGs within the resistome. Proteobacteria predominately harbored ARGs encoding efflux pumps (mean = 4.93 per isolate) and very few encoding antibiotic inactivating enzymes (mean = 0.28 per isolate) whereas the most abundant resistance mechanism in *Bacillus* spp. was antibiotic inactivation (mean = 1.5 per isolate) and the one of the lowest abundance mechanisms was ARGs encoding efflux pumps (mean = 0.03 per isolate).

Within each bacterial genus there were both ARGs unique to one genus and site, such as tet(45) in *Bacillus* from the UD site, and a distinct set of core ARGs that were chromosomally encoded and ubiquitous across thaw levels within a genus, such as *adeF* in *Pseudomonas* (Figure 4a) and *BcII* in *Bacillus* (Figure 4b). The genes unique to one site and taxa, although rare, are more likely accessory determinants that were acquired either through conjugation, transformation, or transduction from other members of the soil community and are therefore more of an interest in terms of clinical risk. The more widespread core genes are likely a result of clonal expansion and less prone to horizontal gene transfer compared to the aforementioned accessory determinants associated with genomic hotspots and mobile genetic elements such as integrons, plasmids, transposons [43,44]. Yet core resistance genes in soil bacteria still pose a risk because they have the potential to be mobilized through transformation or transduction and are widespread within taxa as an intrinsic part of the genome that likely plays a role in both the colonization of the rhizosphere and high-level antibiotic resistance associated with many environmental borne opportunistic pathogens such as *Pseudomonas aeruginosa* [45,46]. 

Some of the isolates in this study do in fact belong to taxa of known opportunistic human pathogens, such as *Pantonea agglomerans*, *Bacillus cereus*, and several *Pseudomonas* spp. and were found to carry both chromosomally encoded and plasmid-borne ARGs [47,48]. However, even nonpathogenic soil bacteria regularly interact with waterways, air, and built habitats, such as hospital surfaces generating a potential for HGT from one biosphere to another. When exposed to antibiotics, even the nonpathogenic commensal bacteria carrying resistance determinants acquired from environmental sources can be selected for promoting the clonal expansion and increased risk for spread of ARGs to the pathogenic bacteria the antibiotic is targeting [49]. A study by Hu et al. 2016 analyzed the mobilome of 23,425 bacterial genomes and found that mobile ARGs are mainly present in four bacterial phyla, the top two of which were *Proteobacteria* (399 mobile ARGs) and *Firmicutes* (86 mobile ARGs) [23]. All of the FPES isolates belong to these two phyla and six isolates were shown to carry plasmid-borne ARGs. Although only 1.6% of the ARGs copies from FPES isolates were located on plasmids, presence on these MGEs is telling of the low, but real, potential for ARGs from these soils to be disseminated. Moreover the higher number of plasmid-borne ARGs in FPES isolates compared to the RefSoil+ bacteria from the same genera, suggests the role local soil attributes have in selecting for plasmid-carriage further highlighting the clinical significance of ARGs harbored in Alaskan soils.

We found ARGs with high sequence identity and full-length coverage to those present in the CARD database. This presence of highly homologous ARGs highlights that resistance determinants in soils can be similar to those in clinical settings, rather than just ancient divergent homologs. However, the mean overall percent identity of ARGs in our isolates (68.4% ± 19.) suggests that many of the ARGs identified in our isolates are novel homologs. The most abundant ARG encoding antibiotic inactivating enzymes, fosB, had a high mean percent identity and full-length gene coverage to fosB genes in the CARD database. This gene encodes fosfomycin thiol transferase that confers resistance to an antibiotic derived from secondary metabolites produced by soil-dwelling bacteria including *Streptomyces* and *pseudomonads* [42]. Both *Streptomyces* and *pseudomonads* have been found to be abundant in FPES metagenomic datasets [24] highlighting the taxonomic potential for the production of fosfomycin at this locale. This potential along with the high abundance of fosB found in this study is suggestive of the selective advantage encoding antibiotic inactivating enzymes may have in competing against antibiotic producing bacterial taxa in the soil community.

In our soil isolates genes encoding efflux pumps were the most abundant with resistance-nodulation-cell division (RND) efflux pumps being the most abundant genefamily found in all *Proteobacteria* isolates. RND efflux pumps have been described as a major tolerance mechanism allowing the effective extrusion of organic solvent from the interior of the cell to the exterior environment, this mechanism is found to be especially prevalent in *Pseudomonas* [50]. As environmental change increasingly affects the arctic in the form of higher annual ambient air temperature and anthropogenic disturbance of soils, microbial life within the active layer have to cope with the release of biogenic volatile organic compounds (BVOC) amplified by thawing permafrost [51]. Efflux pumps could provide an effective mechanism for coping with toxic substances, such as BVOCs that will increase in concentration within active layer soils with permafrost thaw and antibiotics.

### Limitations

There are a variety of published antibiotic resistance gene databases used for the annotation of resistance genes. These resources are often created through the curation of genes identified in the scientific literature, and only contain functional annotations for genes with published experimental data. Results from annotation of these databases can be reflective of the database chosen due to inequality across gene and protein annotation resources [52]. Moreover, since antibiotic resistance is more commonly analyzed in clinical situations, genes in this database can be biased towards clinical phylogenies and are restricted to known resistance determinants thus missing novel resistant determinants in environmental communities that could be identified via functional vector based tests [53].

One of the most widely used ARG databases is the Comprehensive Antibiotic Resistance Database (CARD) [54]. The CARD database provides well-developed and extensive antibiotic resistance ontology (ARO) and monthly curation updates to include the most up to date ARG reference data which is exclusively derived from peer-reviewed publications validated by clinical or experimental data [55]. A 2016 study found that CARD was able to outperform other popular AR databases including ARDB, ResFinder, and CBMAR by correctly identifying down to a variant level for all variants of the two genes tested, bla_VIM_ and bla_NDM_, and unlike any of the other databases was able to accurately identify the maximum number of resistance genes from the whole genome sequences of 3 strains of methicillin resistant *Staphylococcus aureus* [56]. Based on these findings, we decided to use CARD for the annotation of our isolates in this study.

Another limitation of this study is that we cannot attribute differences between sampling sites specifically to a single factor such as permafrost thaw, vegetation shifts, or soil characteristics. Rather we attributed site level differences overall, which is a culmination of these biotic and abiotic factors present at FPES. This study does not provide direct evidence of a horizontal gene transfer event from soil bacteria to pathogenic bacteria; however, we do identify the remarkable abundance and diversity of antibiotic resistance in Alaskan soils. The resistance genes identified here are a restricted representation of the Alaskan soil community because this study is limited in geography, to the CARD ARG database, and to the culturable nonfastidious aerobic to facultative anaerobic bacteria of the phyla *Proteobacteria* and *Firmicutes*. Despite this restricted scope, the abundance, diversity, and presence of ARGs on plasmids is suggestive of the extent these soils have as a reservoir for antibiotic resistance and for the potential to compromise health. Furthermore the over-representation of Proteobacteria in disturbed cores and *Bacillales* in undisturbed cores revealed from previous metagenomic analyses of FPES soils parallels the significant difference we observed in the number of ARGs per isolate from MD and SD sites driven by the shifting proportion of Firmicutes and Proteobacteria.

## 5. Conclusions

In this study, we unearthed antibiotic resistance in bacteria from active layer soils of Interior Alaska associated with permafrost thaw. Most bacterial isolates from this locale were phenotypically resistant to clinically significant antibiotics and encoded both highly homologous and divergent homologs to previously identified resistance determinants. We even identified several resistance genes on plasmids, highlighting the risk these soils have in the dissemination of antibiotic resistance. The significant difference in ARG abundance between the most disturbed and undisturbed isolates driven by a difference in the isolate’s phylogeny, along with previously identified enrichment in Proteobacteria at this site, highlights how community shifts with thaw may enrich for the taxa that increase the abundance of resistance genes comprising the resistome. When compared to the genomes of soil bacteria from a global database, RefSoil+, there were differences in the number of ARGs per isolate and a higher abundance of plasmid-borne ARGs in our isolates from equivalent genera that emphasized how local biotic and abiotic factors shape fine scale differences in the resistance profiles. Moreover, the high-quality whole genome assemblies generated in this study can be used for future analyses into diverse areas of research, such as the coexistence of virulence factors and antibiotic resistance [57], the genomics of cold adaptation of psychrophilic microorganisms [58], and more in depth analyses into the mobile genetic elements that have the potential to propagate the spread of resistance. As antibiotic resistance continues to emerge and rapidly spread in clinical settings, studies like this will be imperative for building insight into the ecology of environmental resistance genes in order to understand the threat they pose to human health.

## Figures and Tables

**Table 1:** Number of isolates within each phylum and genus by Fairbanks Permafrost Experiment Station (FPES) site.

Taxonomy		FPES Site			
Phylum	Genus	UD	SD	MD	Total
Firmicute	*Bacillus*	14	11	5	30
Firmicute	*Exiguobacterium*	0	0	1	1
Proteobacteria	*Erwinia*	1	1	3	5
Proteobacteria	*Pantoea*	0	0	2	2
Proteobacteria	*Pseudomonas*	14	18	19	51
Proteobacteria	*Serratia*	1	0	0	1
	**Total**	30	30	30	90

UD = Undisturbed, SD = Semi-Disturbed, MD = Most-Disturbed.

**Table 2:** List of ARGs found on plasmids in FPES Isolates along with description of each ARG’s resistance mechanism, drug class, gene family, and FPES host taxa.

PLASMID BORNE ARGs IN FPES ISOLATES					
Best Hit ARO	Resistance Mechanism	Drug Class	AMR Gene Family	Genus Origin	Count
BES-1	antibiotic inactivation	carbapenem; cephalosporin; penam	SIM beta-lactamase	*Pantoea*	1
bcrC	antibiotic target alteration	peptide antibiotic	udecaprenyl pyrophosphate related proteins	*Bacillus*	2
TriC	antibiotic efflux	triclosan	RND antibiotic efflux pump	*Pseudomonas*	2
KpnF	antibiotic efflux	Broad Spectrum	MFS antibiotic efflux pump	*Erwinia*	1

## Data Availability

This genome project is indexed at GenBank under BioProject accession numbers PRJNA684363, PRJNA486198, PRJNA486356, PRJNA525875. These whole genomes have been deposited in GenBank under the accession nos. SAMN09840060, SAMN09843991, SAMN11079031, SAMN17054779-17054865.

## Supplementary Materials

The following are available online at https://www.mdpi.com/2076-2607/9/1/116/s1, Table S1: ONT library prep, Table S2: Assembly Statistics, Table S3: CARD Hit Descriptions, Table S4: Isolate resistance phenotypic and genomic results overview.

## Abbreviations

ARG	antibiotic resistance gene
ARO	antibiotic resistance ontology
BVOC	biogenic volatile organic compounds
CARD	Comprehensive Antibiotic Resistance Database
CLSI	Clinical and Laboratory Standards Institute
FPES	Fairbanks Permafrost Experiment Station
HGT	horizontal gene transfer
MD	Most-disturbed
MGE	mobile genetic element
ONT	Oxford Nanopore Technologies
RGI	Resistance Gene Identifier
RND	Resistance-nodulation-cell division
SD	Semidisturbed
TSB	Tryptic Soy Broth
UD	Undisturbed

## Appendix A

**Table A1:** Zone of inhibition breakpoints (mm) from the US Clinical and Laboratory Standards Institute M100, 30th ed. based on the genus of FPES isolates against Ampicillin, Chloramphenicol, Erythromycin, Kanamycin, and Tetracycline with R = resistant, I = intermediate, and S = susceptible.

CLSI Breakpoint Class	FPES Isolate Genus	Ampicillin			Chloramphenicol			Erythromycin			Kanamycin			Tetracycline		
		R	I	S	R	I	S	R	I	S	R	I	S	R	I	S
*Pseudomonas* spp.	*Pseudomonas*	−	−	−	12	13–17	18	−	−	−	12	13–14	15	11	12–14	15
Enterobacterales	*Erwinia*	13	14–16	17	12	13–17	18	12	−	13	13	14–17	18	11	12–14	15
Enterobacterales	*Pantonea*	13	14–16	17	12	13–17	18	12	−	13	13	14–17	18	11	12–14	15
Enterobacterales	*Serratia*	13	14–16	17	12	13–17	18	12	−	13	13	14–17	18	11	12–14	15
*Enterococcus* spp.	*Bacillus*	16	−	17	12	13–17	18	13	14–22	23	−	−	−	14	15–18	19
*Enterococcus* spp.	*Exiguobacterium*	16	−	17	12	13–17	18	13	14–22	23	−	−	−	14	15–18	19
