# Supplementary material for: Unearthing Antibiotic Resistance Associated with Disturbance-Induced Permafrost Thaw in Interior Alaska
Source: Microorganisms. 2021 Jan 6;9(1):116. doi: 10.3390/microorganisms9010116 (PMC7825290; doi:10.3390/microorganisms9010116)
Supplement: Supplementary file 1 [file microorganisms-09-00116-s001.pdf]

Supplemental Materials.

Table S1. ONT library prep and sequencing run by isolate

Table S2. Assembly statistics and checkM metrics of quality for each isolate's assembly.

Table S3. Description of CARD gene hits from all isolates in terms of resistance mechanism, best hit gene, drug class associated with that gene, gene family, count of gene copies for each hit across the 90 isolates, along with the mean and standard deviation of percent length of reference sequence and percent identity as defined by RGI.

Table S4. Number of copies of each gene hit in the sequence data by isolate and phenotypic results with zone of inhibition measurements (mm) and CLSI breakpoint interpretation (R-resistant; I-Intermediate; S-susceptible; NA- not applicable for due to intrinsic resistance) for Kirby-Bauer disk diffusion assay.

Table S1. ONT library prep and sequencing run by isolate

ONT Libarary Prep	Sequencing Date	Run Script	RunTime	Samples Sequenced											
SQK-RBK004	20180724A	RBK004	48hr	TH01	TH10	TH13	TH21	TH31	TH33	TH45	TH52	TH61	TH64	TH72	TH80
	20180724B	RBK004	48hr	TH04	TH12	TH15	TH30	TH34	TH35	TH41	TH56	TH65	TH66	TH74	TH86
	20180724C	RBK004	48hr	TH03	TH18	TH19	TH29	TH39	TH40	TH47	TH60	TH69	TH76	TH77	TH90
	20200203	RBK004	72hr	TH02	TH11	TH22	TH32	TH42	TH51	TH62	TH73	TH82	TH05	TH43	TH83
	20200207	RBK004	72hr	TH08	TH58	TH50	TH14	TH16							
	20200214	RBK004	72hr	TH63	TH36	TH06	TH75	TH44	TH84	TH53	TH23	TH67	TH54		
	20200221	RBK004	72hr	TH07	TH17	TH24	TH25	TH37	TH46	TH55	TH68	TH71	TH85	TH87	
	20200224	RBK004	72hr	TH70	TH38	TH10	TH78	TH48	TH20	TH86	TH57	TH27	TH15	TH49	
VSK-VSK002	20180505	VSK002	48hr	TH81											
	20180515	VSK002	48hr	TH59											
	20190228	VSK002	48hr	TH26											
VSK- VMK002	20191113	VMK002	72hr	TH28											

Table S2. Assembly statistics and checkM metrics of quality for each isolate's assembly.

Isolate	length	Contigs	Mean contig length	largest contig	N50	N50,n	GC %	GC std	Coding density:	# predicted genes:	Completeness:	Contamination:	check_M marker lineage	BioSample Accession
TH01	7289415	1	7289415	7289415	7289415	1	62%	0.00	0.89	6521	100	0.87	o__Pseudomonadales	SAMN17054779
TH02	6428555	1	6428555	6428555	6428555	1	59%	0.00	0.87	5729	100	0.05	g__Pseudomonas	SAMN17054780
TH03	6292986	158	39829	230652	56073	39	59%	0.02	0.88	5752	85	1.69	o__Pseudomonadales	SAMN17054781
TH04	6270276	2	3135138	6269627	6269627	1	60%	0.00	0.89	5593	100	0.52	g__Pseudomonas	SAMN17054782
TH05	7101889	70	101456	491789	165760	13	62%	0.02	0.89	6507	99	0.87	o__Pseudomonadales	SAMN17054783
TH06	6565344	4	1641336	5329916	5329916	1	59%	0.00	0.88	5759	100	0.34	g__Pseudomonas	SAMN17054784
TH07	7282377	2	3641189	7270375	7270375	1	62%	0.00	0.89	6530	100	0.87	o__Pseudomonadales	SAMN17054785
TH08	6565193	6	1094199	2115574	1462546	2	59%	0.00	0.88	5865	100	0.34	g__Pseudomonas	SAMN17054786
TH09	6300396	72	87506	1275846	563232	4	60%	0.04	0.89	5737	100	0.19	o__Pseudomonadales	SAMN17054787
TH10	4977657	179	27808	105294	35424	52	59%	0.02	0.87	5195	75	0.76	g__Pseudomonas	SAMN17054788
TH11	3239959	138	23478	116995	30084	34	36%	0.02	0.81	4199	80	0.27	o__Bacillales	SAMN17054789
TH12	5819177	6	969863	5081309	5081309	1	35%	0.03	0.82	5863	99	0.50	g__Bacillus	SAMN17054790
TH13	6132576	25	245303	4565714	4565714	1	35%	0.04	0.83	6375	99	0.76	g__Bacillus	SAMN17054791
TH14	6352696	3	2117565	5830573	5830573	1	38%	0.00	0.78	6056	99	3.44	f__Bacillaceae	SAMN17054792
TH15	6252465	2	3126233	5280705	5280705	1	59%	0.00	0.87	5674	100	0.11	o__Pseudomonadales	SAMN17054793
TH16	6232267	12	519356	2999140	2358609	2	38%	0.03	0.78	5927	99	3.72	f__Bacillaceae	SAMN17054794
TH17	5747064	6	957844	5159847	5159847	1	35%	0.03	0.82	5826	98	0.77	g__Bacillus	SAMN17054795
TH18	7152072	1	7152072	7152072	7152072	1	60%	0.00	0.88	6271	100	0.54	o__Pseudomonadales	SAMN17054796
TH19	5767003	16	360438	2590843	2009945	2	35%	0.03	0.82	5822	99	0.29	g__Bacillus	SAMN17054797
TH20	6987576	201	34764	277831	70357	26	59%	0.02	0.88	6383	99	0.24	o__Pseudomonadales	SAMN17054798
TH21	6760171	2	3380086	6753006	6753006	1	62%	0.11	0.89	6000	100	0.41	g__Pseudomonas	SAMN17054799
TH22	5822214	3	1940738	5423614	5423614	1	35%	0.02	0.82	5874	98	1.55	g__Bacillus	SAMN17054800
TH23	5806530	7	829504	3853346	3853346	1	35%	0.03	0.82	5907	98	1.55	g__Bacillus	SAMN17054801
TH24	6188561	5	1237712	5688067	5688067	1	38%	0.02	0.78	5938	98	3.17	f__Bacillaceae	SAMN17054802
TH25	5862716	39	150326	1409789	711156	3	35%	0.02	0.82	5916	98	0.95	g__Bacillus	SAMN17054803
TH26	5790936	2	2895468	5307118	5307118	1	35%	0.01	0.82	5779	99	0.15	g__Bacillus	SAMN11079031
TH27	6276694	4	1569174	5739858	5739858	1	38%	0.02	0.78	5984	99	3.17	f__Bacillaceae	SAMN17054804
TH28	5123563	239	21438	170590	39071	37	55%	0.04	0.88	4859	98	0.89	f__Enterobacteriaceae	SAMN17054805
TH29	3154365	1477	2136	11893	2306	442	54%	0.04	0.87	4077	72	0.55	f__Enterobacteriaceae	SAMN17054806
TH30	5897091	6	982849	5270848	5270848	1	35%	0.04	0.82	5863	99	0.21	g__Bacillus	SAMN17054807
TH31	7196786	38	189389	950737	452287	6	59%	0.03	0.86	7112	98	1.69	g__Pseudomonas	SAMN17054808
TH32	7208438	4	1802110	6375311	6375311	1	61%	0.03	0.89	6670	100	0.27	g__Pseudomonas	SAMN17054809
TH33	6929409	1	6929409	6929409	6929409	1	61%	0.00	0.89	6258	100	0.83	g__Pseudomonas	SAMN17054810
TH34	7008149	35	200233	752473	447628	6	60%	0.02	0.89	6412	100	0.21	g__Pseudomonas	SAMN17054811
TH35	6185610	409	15124	421554	141290	14	61%	0.05	0.88	5996	98	0.57	o__Pseudomonadales	SAMN17054812
TH36	6757750	1	6757750	6757750	6757750	1	58%	0.00	0.89	6241	100	0.62	o__Pseudomonadales	SAMN17054813
TH37	6297323	357	17640	345118	170805	13	61%	0.05	0.88	6060	100	0.57	o__Pseudomonadales	SAMN17054814
TH38	6881557	56	122885	870092	341859	6	59%	0.02	0.89	6255	99	0.75	g__Pseudomonas	SAMN17054815
TH39	7179220	8	897403	2756366	1664454	2	59%	0.01	0.88	6562	100	1.08	g__Pseudomonas	SAMN17054816
TH40	6136442	221	27767	225211	73707	29	61%	0.04	0.87	6090	97	0.68	o__Pseudomonadales	SAMN17054817
TH41	6782636	9	753626	3093317	1191987	2	59%	0.00	0.87	6095	99	1.17	g__Pseudomonas	SAMN17054818
TH42	6581347	1	6581347	6581347	6581347	1	60%	0.00	0.87	5801	100	0.97	g__Pseudomonas	SAMN17054819
TH43	6500574	5	1300115	2489632	1546032	2	59%	0.00	0.87	5811	100	0.11	g__Pseudomonas	SAMN17054820
TH44	5938642	59	100655	1212980	347271	5	35%	0.03	0.81	6211	97	1.55	g__Bacillus	SAMN17054821
TH45	5862293	15	390820	4124331	4124331	1	35%	0.05	0.82	5984	98	1.86	g__Bacillus	SAMN17054822
TH46	6582152	1	6582152	6582152	6582152	1	60%	0.00	0.87	5804	100	0.97	g__Pseudomonas	SAMN17054823
TH47	6580544	1	6580544	6580544	6580544	1	60%	0.00	0.87	5800	100	0.97	g__Pseudomonas	SAMN17054824
TH48	6751879	1	6751879	6751879	6751879	1	62%	0.00	0.89	5973	100	0.14	g__Pseudomonas	SAMN17054825
TH49	6641420	2	3320710	3792784	3792784	1	60%	0.00	0.87	5906	100	0.40	g__Pseudomonas	SAMN17054826
TH50	5932849	8	741606	3087957	3087957	1	35%	0.04	0.82	6012	98	1.86	g__Bacillus	SAMN17054827
TH51	5966177	6	994363	3086880	3086880	1	35%	0.04	0.82	6163	98	1.55	g__Bacillus	SAMN17054828
TH52	5965749	15	397717	1979436	1731552	2	35%	0.02	0.82	6074	99	0.61	g__Bacillus	SAMN17054829
TH53	5954056	5	1190811	5462858	5462858	1	35%	0.04	0.82	6117	98	1.53	g__Bacillus	SAMN17054830
TH54	5941371	4	1485343	5495595	5495595	1	35%	0.08	0.82	6055	98	1.55	g__Bacillus	SAMN17054831
TH55	4877094	637	7656	159431	12721	90	36%	0.02	0.85	5058	94	0.38	g__Bacillus	SAMN17054832
TH56	4853631	1	4853631	4853631	4853631	1	55%	0.00	0.88	4388	100	0.00	f__Enterobacteriaceae	SAMN17054833
TH57	5203616	617	8434	188393	15558	81	36%	0.02	0.85	5407	97	1.48	g__Bacillus	SAMN17054834
TH58	5992248	9	665805	2344683	2197132	2	35%	0.03	0.82	6107	99	1.86	g__Bacillus	SAMN17054835
TH59	4906365	790	6211	80706	10625	111	36%	0.02	0.86	5137	94	0.73	g__Bacillus	SAMN17054836
TH60	6581323	1	6581323	6581323	6581323	1	60%	0.00	0.87	5795	100	0.97	g__Pseudomonas	SAMN17054837
TH61	6408248	1	6408248	6408248	6408248	1	59%	0.00	0.88	5691	100	0.05	g__Pseudomonas	SAMN17054838
TH62	6683507	3	2227836	6638173	6638173	1	59%	0.06	0.88	6108	100	0.28	o__Pseudomonadales	SAMN17054839
TH63	6157383	1	6157383	6157383	6157383	1	59%	0.00	0.87	5566	99	0.79	g__Pseudomonas	SAMN17054840
TH64	6733090	2	3366545	6465401	6465401	1	59%	0.05	0.88	6231	100	0.30	o__Pseudomonadales	SAMN17054841
TH65	6397841	1	6397841	6397841	6397841	1	60%	0.00	0.88	5882	100	0.66	g__Pseudomonas	SAMN17054842
TH66	6131349	144	42579	201108	70924	28	59%	0.02	0.88	5639	98	14.04	k__Bacteria	SAMN17054843
TH67	6682590	3	2227530	6637397	6637397	1	59%	0.06	0.88	6102	100	0.28	o__Pseudomonadales	SAMN17054844
TH68	7157743	3	2385914	4088561	4088561	1	59%	0.03	0.87	6529	100	0.21	o__Pseudomonadales	SAMN17054845
TH69	5914792	1	5914792	5914792	5914792	1	59%	0.00	0.88	5243	100	0.08	g__Pseudomonas	SAMN17054846
TH70	7078794	3	2359598	6999391	6999391	1	60%	0.01	0.88	6367	100	1.09	o__Pseudomonadales	SAMN17054847
TH71	6270423	38	165011	1061543	465089	5	59%	0.03	0.88	5540	97	0.15	g__Pseudomonas	SAMN17054848
TH72	7744191	23	336704	3545502	1844773	2	59%	0.05	0.87	6963	99	0.67	g__Pseudomonas	SAMN17054849
TH73	3185552	5	637110	3152340	3152340	1	48%	0.09	0.89	3267	99	0.33	c__Bacilli	SAMN17054850
TH74	6766338	6	1127723	2599615	2167177	2	60%	0.03	0.87	6131	100	1.07	g__Pseudomonas	SAMN17054851
TH75	6765294	3	2255098	6708000	6708000	1	60%	0.04	0.87	6128	100	1.07	g__Pseudomonas	SAMN17054852
TH76	5908038	1	5908038	5908038	5908038	1	63%	0.00	0.89	5248	100	0.14	o__Pseudomonadales	SAMN17054853
TH77	7361083	8	920135	6195251	6195251	1	60%	0.05	0.88	6784	100	0.87	g__Pseudomonas	SAMN17054854
TH78	5908405	1	5908405	5908405	5908405	1	63%	0.00	0.89	5221	100	0.14	o__Pseudomonadales	SAMN17054855
TH79	4583151	71	64551	283231	114121	14	56%	0.03	0.88	4203	100	0.24	f__Enterobacteriaceae	SAMN17054856
TH80	6539415	7	934202	3216008	1092864	2	59%	0.01	0.88	5775	100	0.15	g__Pseudomonas	SAMN17054857
TH81	4984484	4	1246121	4128832	4128832	1	55%	0.03	0.85	4539	100	1.50	f__Enterobacteriaceae	SAMN09840060
TH82	4647952	2	2323976	4554121	4554121	1	56%	0.01	0.88	4269	100	0.24	f__Enterobacteriaceae	SAMN17054858
TH83	3297868	60	54964	369722	114945	8	66%	0.21	0.84	3993	89	18.46	k__Bacteria	SAMN17054859
TH84	4930048	54	91297	520953	141253	11	55%	0.03	0.85	4540	100	1.80	f__Enterobacteriaceae	SAMN17054860
TH85	5873192	6	978865	5302587	5302587	1	35%	0.03	0.82	5896	99	0.32	g__Bacillus	SAMN17054861
TH86	5476598	280	19559	240496	45743	29	35%	0.03	0.84	5642	98	0.77	g__Bacillus	SAMN17054862
TH87	5967729	13	459056	2640400	2484126	2	35%	0.03	0.82	6009	99	0.48	g__Bacillus	SAMN17054863
TH88	4903410	2	2451705	4824998	4824998	1	55%	0.01	0.88	4444	100	0.05	f__Enterobacteriaceae	SAMN09843991
TH89	5889526	7	841361	5293100	5293100	1	35%	0.03	0.82	6099	99	0.04	g__Bacillus	SAMN17054864
TH90	6751295	1	6751295	6751295	6751295	1	62%	0.00	0.88	6311	99	0.23	g__Pseudomonas	SAMN17054865
**MEAN**	**6096842**	**75**	**2102452**	**3839076**	**3639582**	**13**	**52%**	**0.03**	**0.86**	**5772**	**98**	**1.16**		
**STDER**	**96902**	**22**	**251424**	**261837**	**279870**	**5**	**1%**	**0.00**	**0.00**	**73**	**0**	**0.26**		

Table S3. Description of CARD gene hits from all isolates in terms of resistance mechanism, best hit gene, drug class associated with that gene, gene family, count of gene copies for each hit across the 90 isolates, along with the mean and standard deviation of percent length of reference sequence and percent identity as defined by RGI.

Resistance Mechanism	*Best_Hit_ARO*	*Drug Class*	*AMR Gene Family*	*Count*	*% length of reference sequence*	*% identity*
antibiotic efflux	Acinetobacter baumannii AbaQ	fluoroquinolone antibiotic	major facilitator superfamily (MFS) antibiotic efflux pump	40	101.363 ± 0.109	72.675 ± 0.427
	adeF	fluoroquinolone antibiotic; tetracycline antibiotic	resistance-nodulation-cell division (RND) antibiotic efflux pump	176	99.02 ± 2.902	52.283 ± 11.822
	CRP	macrolide antibiotic; fluoroquinolone antibiotic; penam	RND antibiotic efflux pump	8	100 ± 0	98.87 ± 0.248
	emrR	fluoroquinolone antibiotic	MFS antibiotic efflux pump	7	99.757 ± 0.643	82.049 ± 1.001
	Klebsiella pneumoniae KpnE	Broad Spectrum	MFS antibiotic efflux pump	2	96.67 ± 0	66.95 ± 0
	Klebsiella pneumoniae KpnF	Broad Spectrum	MFS antibiotic efflux pump	8	100 ± 0	73.389 ± 2.903
	Klebsiella pneumoniae KpnH	Broad Spectrum	MFS antibiotic efflux pump	6	99.8 ± 0	86.705 ± 1.38
	msbA	nitroimidazole antibiotic	ABC antibiotic efflux pump	7	100 ± 0	87.827 ± 1.059
	tet(45)	tetracycline antibiotic	MFS antibiotic efflux pump	1	100 ± NA	89.5 ± NA
	TriC	triclosan	RND antibiotic efflux pump	2	2.76 ± 0	100 ± 0
antibiotic inactivation	AAC(6')-32	aminoglycoside antibiotic	AAC(6')	1	122.83 ± NA	100 ± NA
	AAC(6')-Ib7	aminoglycoside antibiotic	AAC(6')	1	65.13 ± NA	100 ± NA
	AAC(6')-Ir	aminoglycoside antibiotic	AAC(6')	3	272.6 ± 0	100 ± 0
	BcII	cephalosporin; penam	Bc beta-lactamase	20	100.39 ± 0	90.755 ± 0.567
	BES-1	penam	BES Beta-lactamase	1	27.05 ± NA	100 ± NA
	BPU-1	penam	BPU Beta-lactamase	4	51.15 ± 0	100 ± 0
	Escherichia coli ampC1 beta-lactamase	cephalosporin; penam	ampC-type beta-lactamase	1	94.7 ± NA	100 ± NA
	Escherichia coli ampH beta-lactamase	cephalosporin; penam	ampC-type beta-lactamase	6	101.733 ± 1.17	70.063 ± 2.367
	FosB	fosfomycin	fosfomycin thiol transferase	25	105.012 ± 6.992	89.055 ± 2.281
antibiotic target alteration	armA	aminoglycoside antibiotic	16S rRNA methyltransferase (G1405)	11	81.214 ± 0.182	100 ± 0
	bcrC	peptide antibiotic	undecaprenyl pyrophosphate related proteins	2	166.995 ± 100.317	100 ± 0
	MCR-4.1	peptide antibiotic	MCR phosphoethanolamine transferase	2	10.63 ± 1.174	100 ± 0
	Morganella morganii gyrB	fluoroquinolone antibiotic	fluoroquinolone resistant gyrB	6	99.75 ± 0	80.43 ± 0.279
	PmrF	peptide antibiotic	pmr phosphoethanolamine transferase	2	100.93 ± 0	80.5 ± 0
	sgm	aminoglycoside antibiotic	16S rRNA methyltransferase	1	16.42 ± NA	100 ± NA
	vanJ	glycopeptide antibiotic	vanJ membrane protein	1	11.52 ± NA	100 ± NA
antibiotic target alteration; antibiotic efflux	Pseudomonas aeruginosa soxR	Broad Spectrum	ABC, MFS, RND antibiotic efflux pump	35	96.793 ± 1.854	68.494 ± 1.961

TableS4.

ISOLATE INFO			Antibiotic Inactivation									Antibiotic Efflux											Antibiotic Target Alteration							Phenotypic Results									
			aminoglycoside			beta-lactams					fosfomycin	fluoroquinolone, tetracyline, macrolide, penam				Broad Spectrum				Nitroimidazole	Tetracycline	Triclosan	aminoglycoside		Peptide antibiotic			fluoroquinolone	glycopeptide	Ampicillan		Chloramphenicol		Erythromyocin		Kanamycin		Tetracycline	
COLONY	ID	GENUS	AAC(6')-32	AAC(6')-Ib7	AAC(6')-Ir	ampC1 beta-lactamase	ampH beta-lactamase	BcII	BES-1	BPU-1	FosB	AbaQ	adeF	CRP	emrR	KpnE	KpnF	soxR	KpnH	msbA	tet(45)	TriC	armA	sgm	bcrC	MCR-4.1	PmrF	gyrB	vanJ	inhibition zone (mm)	Level of Resistance	inhibition zone (mm)	Level of Resistance	inhibition zone (mm)	Level of Resistance	inhibition zone (mm)	Level of Resistance	inhibition zone (mm)	Level of Resistance
UD+4 colony 1	TH01	Pseudomonas	0	0	0	0	0	0	0	0	0	0	3	0	0	0	0	1	0	0	0	0	0	0	0	0	0	0	0	0	NA	10	R	0	NA	17	I	16	I
UD+4 colony 2	TH02	Pseudomonas	0	0	0	0	0	0	0	0	0	1	2	0	0	0	0	1	0	0	0	0	0	0	0	0	0	0	0	0	NA	0	R	0	NA	19	S	14	I
UD+4 colony 3	TH03	Pseudomonas	0	0	0	0	0	0	0	0	0	1	2	0	0	0	0	0	0	0	0	0	1	0	0	0	0	0	0	0	NA	0	R	0	NA	20	S	18	S
UD+4 colony 4	TH04	Pseudomonas	0	0	0	0	0	0	0	0	0	1	3	0	0	0	0	1	0	0	0	0	0	0	0	0	0	0	0	0	NA	0	R	0	NA	10	S	16	I
UD+4 colony 5	TH05	Pseudomonas	0	0	0	0	0	0	0	0	0	0	3	0	0	0	0	1	0	0	0	0	0	0	0	0	0	0	0	0	NA	0	R	0	NA	16	I	15	I
UD+4 colony 6	TH06	Pseudomonas	0	0	0	0	0	0	0	0	0	1	4	0	0	0	0	1	0	0	0	0	0	0	0	0	0	0	0	0	NA	8	R	0	NA	18	I	19	S
UD+4 colony 7	TH07	Pseudomonas	0	0	0	0	0	0	0	0	0	0	3	0	0	0	0	1	0	0	0	0	0	0	0	0	0	0	0	0	NA	0	R	0	NA	15	I	11	I
UD+4 colony 8	TH08	Pseudomonas	0	0	0	0	0	0	0	0	0	1	3	0	0	0	0	1	0	0	0	0	0	0	0	0	0	0	0	0	NA	0	R	0	NA	17	I	21	S
UD+4 colony 9	TH09	Pseudomonas	0	0	0	0	0	0	0	0	0	1	3	0	0	0	0	0	0	0	0	0	0	0	0	0	0	0	0	0	NA	11	R	0	NA	20	S	20	S
UD+4 colony 10	TH10	Pseudomonas	0	1	0	0	0	0	0	0	0	0	1	0	0	0	0	0	0	0	0	0	0	0	0	0	0	0	0	0	NA	0	R	0	NA	18	I	18	S
UD+12 colony 1	TH11	Bacillus	0	0	0	0	0	0	0	0	1	0	0	0	0	0	0	0	0	0	0	0	0	0	0	0	0	0	0	20	S	35	S	35	S	26	S	33	S
UD+12 colony 2	TH12	Bacillus	0	0	0	0	0	0	0	0	1	0	0	0	0	0	0	0	0	0	0	0	0	0	0	0	0	0	0	0	R	30	S	32	S	25	S	26	S
UD+12 colony 3	TH13	Bacillus	0	0	0	0	0	1	0	0	1	0	0	0	0	0	0	0	0	0	0	0	0	0	2	0	0	0	0	0	R	23	S	30	S	20	S	25	S
UD+12 colony 4	TH14	Bacillus	0	0	0	0	0	0	0	0	0	0	0	0	0	0	0	0	0	0	0	0	0	0	0	0	0	0	0	20	S	32	S	30	S	27	S	29	S
UD+12 colony 5	TH15	Pseudomonas	0	0	0	0	0	0	0	0	0	1	3	0	0	0	0	1	0	0	0	0	1	0	0	0	0	0	0	0	NA	13	I	0	NA	14	I	21	S
UD+12 colony 6	TH16	Bacillus	0	0	0	0	0	0	0	0	0	0	0	0	0	0	0	0	0	0	0	0	0	0	0	0	0	0	0	25	S	30	S	33	S	27	S	32	S
UD+12 colony 7	TH17	Bacillus	0	0	0	0	0	1	0	0	1	0	0	0	0	0	0	0	0	0	0	0	0	0	0	0	0	0	0	0	R	40	S	32	S	27	S	25	S
UD+12 colony 8	TH18	Pseudomonas	0	0	0	0	0	0	0	0	0	1	5	0	0	0	0	0	0	0	0	0	1	0	0	0	0	0	0	0	NA	0	R	0	NA	22	S	20	S
UD+12 colony 9	TH19	Bacillus	0	0	0	0	0	1	0	0	1	0	0	0	0	0	0	0	0	0	0	0	0	0	0	0	0	0	0	0	R	30	S	32	S	23	S	24	S
UD+12 colony 10	TH20	Pseudomonas	0	0	0	0	0	0	0	0	0	1	3	0	0	0	0	0	0	0	0	0	1	0	0	0	0	0	0	0	NA	12	I	0	NA	24	S	25	S
UD+20 colony 1	TH21	Erwinia	0	0	0	0	0	0	0	0	0	1	4	0	0	0	0	1	0	0	0	0	0	0	0	0	0	0	0	0	R	31	S	27	S	22	S	28	S
UD+20 colony 2	TH22	Bacillus	0	0	0	0	0	1	0	0	1	0	0	0	0	0	0	0	0	0	0	0	0	0	0	0	0	0	0	0	R	40	S	40	S	32	S	38	S
UD+20 colony 3	TH23	Bacillus	0	0	0	0	0	1	0	0	1	0	0	0	0	0	0	0	0	0	0	0	0	0	0	0	0	0	0	0	R	36	S	30	S	23	S	30	S
UD+20 colony 4	TH24	Bacillus	0	0	0	0	0	0	0	0	0	0	0	0	0	0	0	0	0	0	0	0	0	0	0	0	0	0	0	0	R	37	S	32	S	25	S	29	S
UD+20 colony 5	TH25	Bacillus	0	0	0	0	0	1	0	0	1	0	0	0	0	0	0	0	0	0	1	0	0	0	0	0	0	0	0	0	R	35	S	28	S	24	S	27	S
UD+20 colony 6	TH26	Bacillus	0	0	0	0	0	1	0	0	1	0	0	0	0	0	0	0	0	0	0	0	0	0	0	0	0	0	0	8	R	34	S	32	S	24	S	29	S
UD+20 colony 7	TH27	Bacillus	0	0	0	0	0	0	0	0	0	0	0	0	0	0	0	0	0	0	0	0	0	0	0	0	0	0	0	0	R	37	S	30	S	24	S	30	S
UD+20 colony 8	TH28	Serratia	0	0	0	0	0	0	0	0	0	0	5	1	1	0	1	0	1	1	0	0	0	0	0	0	0	0	0	25	S	36	S	37	S	26	S	32	S
UD+20 colony 9	TH29	Erwinia	0	0	0	0	0	0	0	0	0	0	1	1	1	0	1	0	0	0	0	0	0	0	0	0	0	0	0	0	R	31	S	27	S	22	S	25	S
UD+20 colony 10	TH30	Bacillus	0	0	0	0	0	1	0	0	1	0	0	0	0	0	0	0	0	0	0	0	0	0	0	0	0	0	0	0	R	0	R	0	R	14	I	14	R
SD+4 colony 1	TH31	Pseudomonas	0	0	0	0	0	0	0	0	0	0	2	0	0	0	0	0	0	0	0	0	0	0	0	0	0	0	0	0	NA	0	R	0	NA	23	S	22	S
SD+4 colony 2	TH32	Pseudomonas	0	0	0	0	0	0	0	0	0	1	2	0	0	0	0	1	0	0	0	0	0	0	0	0	0	0	0	0	NA	0	R	0	NA	19	S	16	I
SD+4 colony 3	TH33	Pseudomonas	0	0	0	0	0	0	0	0	0	1	3	0	0	0	0	1	0	0	0	0	0	0	0	0	0	0	0	0	NA	0	R	0	NA	16	I	18	S
SD+4 colony 4	TH34	Pseudomonas	0	0	0	0	0	0	0	0	0	1	3	0	0	0	0	1	0	0	0	0	0	0	0	0	0	0	0	0	NA	0	R	0	NA	11	R	17	I
SD+4 colony 5	TH35	Pseudomonas	0	0	1	0	0	0	0	1	0	0	3	0	0	0	0	0	0	0	0	1	0	0	0	0	0	0	1	0	NA	0	R	0	NA	24	S	19	S
SD+4 colony 6	TH36	Pseudomonas	0	0	0	0	0	0	0	0	0	1	4	0	0	0	0	1	0	0	0	0	1	0	0	0	0	0	0	0	NA	0	R	0	NA	20	S	16	I
SD+4 colony 7	TH37	Pseudomonas	0	0	1	0	0	0	0	1	0	0	3	0	0	0	0	0	0	0	0	1	0	0	0	0	0	0	0	0	NA	0	R	0	NA	20	S	20	S
SD+4 colony 8	TH38	Pseudomonas	0	0	0	0	0	0	0	0	0	1	3	0	0	0	0	1	0	0	0	0	0	0	0	0	0	0	0	0	NA	0	R	0	NA	20	S	20	S
SD+4 colony 9	TH39	Pseudomonas	0	0	0	0	0	0	0	0	0	2	3	0	0	0	0	1	0	0	0	0	0	0	0	0	0	0	0	0	NA	0	R	0	NA	23	S	20	S
SD+4 colony 10	TH40	Pseudomonas	0	0	1	0	0	0	0	1	0	0	2	0	0	0	0	0	0	0	0	0	0	0	0	0	0	0	0	0	NA	0	R	0	NA	20	S	20	S
SD+12 colony 1	TH41	Pseudomonas	0	0	0	0	0	0	0	0	0	1	3	0	0	0	0	1	0	0	0	0	1	0	0	0	0	0	0	9	NA	19	S	11	NA	9	R	14	I
SD+12 colony 2	TH42	Pseudomonas	0	0	0	0	0	0	0	0	0	1	5	0	0	0	0	1	0	0	0	0	0	0	0	0	0	0	0	0	NA	0	R	0	NA	9	R	14	I
SD+12 colony 3	TH43	Pseudomonas	0	0	0	0	0	0	0	0	0	1	2	0	0	0	0	1	0	0	0	0	0	0	0	0	0	0	0	0	NA	0	R	0	NA	18	I	16	I
SD+12 colony 4	TH44	Bacillus	0	0	0	0	0	1	0	0	1	0	0	0	0	0	0	0	0	0	0	0	0	0	0	0	0	0	0	0	R	28	S	24	S	21	S	26	S
SD+12 colony 5	TH45	Bacillus	0	0	0	0	0	1	0	0	1	0	0	0	0	0	0	0	0	0	0	0	0	0	0	0	0	0	0	0	R	28	S	24	S	20	S	24	S
SD+12 colony 6	TH46	Pseudomonas	0	0	0	0	0	0	0	0	0	1	5	0	0	0	0	1	0	0	0	0	0	0	0	0	0	0	0	0	NA	0	R	0	NA	15	I	15	I
SD+12 colony 7	TH47	Pseudomonas	0	0	0	0	0	0	0	0	0	1	5	0	0	0	0	1	0	0	0	0	0	0	0	0	0	0	0	0	NA	0	R	0	NA	15	I	14	I
SD+12 colony 8	TH48	Pseudomonas	0	0	0	0	0	0	0	0	0	1	4	0	0	0	0	1	0	0	0	0	0	0	0	0	0	0	0	0	NA	0	R	0	NA	17	I	11	R
SD+12 colony 9	TH49	Pseudomonas	0	0	0	0	0	0	0	0	0	1	4	0	0	0	0	1	0	0	0	0	0	0	0	0	0	0	0	0	NA	11	R	0	NA	16	I	21	S
SD+12 colony 10	TH50	Bacillus	0	0	0	0	0	1	0	0	1	0	0	0	0	0	0	0	0	0	0	0	0	0	0	0	0	0	0	0	R	31	S	30	S	23	S	25	S
SD+20 colony 1	TH51	Bacillus	0	0	0	0	0	1	0	0	1	0	0	0	0	0	0	0	0	0	0	0	0	0	0	0	0	0	0	10	R	31	S	29	S	28	S	30	S
SD+20 colony 2	TH52	Bacillus	0	0	0	0	0	0	0	0	1	0	0	0	0	0	0	0	0	0	0	0	0	0	0	0	0	0	0	0	R	31	S	24	S	22	S	22	S
SD+20 colony 3	TH53	Bacillus	0	0	0	0	0	1	0	0	1	0	0	0	0	0	0	0	0	0	0	0	0	0	0	0	0	0	0	0	R	29	S	30	S	20	S	22	S
SD+20 colony 4	TH54	Bacillus	0	0	0	0	0	1	0	0	1	0	0	0	0	0	0	0	0	0	0	0	0	0	0	0	0	0	0	24	S	33	S	34	S	27	S	30	S
SD+20 colony 5	TH55	Bacillus	0	0	0	0	0	0	0	0	1	0	0	0	0	0	0	0	0	0	0	0	0	0	0	1	0	0	0	0	R	32	S	28	S	24	S	15	I
SD+20 colony 6	TH56	Erwinia	0	0	0	0	1	0	0	0	0	0	3	1	1	0	1	0	1	1	0	0	0	0	0	0	0	1	0	0	R	29	S	30	S	24	S	24	S
SD+20 colony 7	TH57	Bacillus	0	0	0	0	0	1	0	0	1	0	0	0	0	0	0	0	0	0	0	0	0	1	0	1	0	0	0	23	S	30	S	35	S	24	I	30	S
SD+20 colony 8	TH58	Bacillus	0	0	0	0	0	1	0	0	1	0	0	0	0	0	0	0	0	0	0	0	0	0	0	0	0	0	0	0	R	14	I	0	R	16	S	16	I
SD+20 colony 9	TH59	Bacillus	0	0	0	0	0	0	0	0	1	0	0	0	0	0	0	0	0	0	0	0	0	0	0	0	0	0	0	0	R	31	S	27	S	22	S	24	S
SD+20 colony 10	TH60	Pseudomonas	0	0	0	0	0	0	0	0	0	1	5	0	0	0	0	1	0	0	0	0	0	0	0	0	0	0	0	0	NA	32	S	30	NA	25	S	25	S
MD+4 colony 1	TH61	Pseudomonas	0	0	0	0	0	0	0	0	0	1	3	0	0	0	0	0	0	0	0	0	0	0	0	0	0	0	0	0	NA	0	R	0	NA	12	R	19	S
MD+4 colony 2	TH62	Pseudomonas	0	0	0	0	0	0	0	0	0	1	3	0	0	0	0	1	0	0	0	0	0	0	0	0	0	0	0	0	NA	0	R	0	NA	18	I	16	I
MD+4 colony 3	TH63	Pseudomonas	0	0	0	0	0	0	0	0	0	1	3	0	0	0	0	0	0	0	0	0	1	0	0	0	0	0	0	0	NA	0	R	0	NA	20	S	18	S
MD+4 colony 4	TH64	Pseudomonas	0	0	0	0	0	0	0	0	0	1	3	0	0	0	0	1	0	0	0	0	0	0	0	0	0	0	0	0	NA	0	R	0	NA	19	S	15	I
MD+4 colony 5	TH65	Pseudomonas	0	0	0	0	0	0	0	0	0	1	2	0	0	0	0	1	0	0	0	0	0	0	0	0	0	0	0	0	NA	0	R	0	NA	20	S	21	S
MD+4 colony 6	TH66	Pseudomonas	0	0	0	0	0	0	0	0	0	0	2	0	0	0	0	1	0	0	0	0	0	0	0	0	0	0	0	0	NA	0	R	0	NA	20	S	20	S
MD+4 colony 7	TH67	Pseudomonas	0	0	0	0	0	0	0	0	0	1	3	0	0	0	0	1	0	0	0	0	0	0	0	0	0	0	0	0	NA	0	R	0	NA	16	I	15	I
MD+4 colony 8	TH68	Pseudomonas	1	0	0	0	0	0	0	1	0	1	3	0	0	0	0	1	0	0	0	0	0	0	0	0	0	0	0	0	NA	0	R	0	NA	22	S	25	S
MD+4 colony 9	TH69	Pseudomonas	0	0	0	0	0	0	0	0	0	1	2	0	0	0	0	1	0	0	0	0	0	0	0	0	0	0	0	0	NA	0	R	0	NA	15	I	15	I
MD+4 colony 10	TH70	Pseudomonas	0	0	0	0	0	0	0	0	0	1	3	0	0	0	0	1	0	0	0	0	1	0	0	0	0	0	0	0	NA	0	R	0	NA	20	S	16	I
MD+12 colony 1	TH71	Pseudomonas	0	0	0	0	0	0	0	0	0	1	2	0	0	0	0	1	0	0	0	0	1	0	0	0	0	0	0	0	NA	8	R	0	NA	19	S	12	I
MD+12 colony 2	TH72	Pseudomonas	0	0	0	0	0	0	0	0	0	1	5	0	0	0	0	0	0	0	0	0	1	0	0	0	0	0	0	0	NA	0	R	0	NA	22	S	18	S
MD+12 colony 3	TH73	Exiguobacterium	0	0	0	0	0	0	0	0	0	0	0	0	0	0	0	0	0	0	0	0	0	0	0	0	0	0	0	27	S	27	S	26	S	21	S	31	S
MD+12 colony 4	TH74	Pseudomonas	0	0	0	0	0	0	0	0	0	1	3	0	0	0	0	0	0	0	0	0	0	0	0	0	0	0	0	0	NA	11	R	0	NA	21	S	21	S
MD+12 colony 5	TH75	Pseudomonas	0	0	0	0	0	0	0	0	0	1	3	0	0	0	0	0	0	0	0	0	0	0	0	0	0	0	0	0	NA	9	R	0	NA	20	S	19	S
MD+12 colony 6	TH76	Pseudomonas	0	0	0	0	0	0	0	0	0	0	2	0	0	0	0	0	0	0	0	0	0	0	0	0	0	0	0	0	NA	0	R	0	NA	17	I	14	I
MD+12 colony 7	TH77	Pseudomonas	0	0	0	1	0	0	0	0	0	1	3	0	0	0	0	1	0	0	0	0	0	0	0	0	0	0	0	0	NA	0	R	0	NA	14	I	18	S
MD+12 colony 8	TH78	Pseudomonas	0	0	0	0	0	0	0	0	0	0	2	0	0	0	0	0	0	0	0	0	0	0	0	0	0	0	0	0	NA	0	R	0	NA	16	I	13	I
MD+12 colony 9	TH79	Erwinia	0	0	0	0	1	0	0	0	0	0	2	1	1	0	1	0	1	1	0	0	0	0	0	0	1	1	0	9	R	18	I	9	R	16	I	15	I
MD+12 colony 10	TH80	Pseudomonas	0	0	0	0	0	0	0	0	0	1	3	0	0	0	0	1	0	0	0	0	1	0	0	0	0	0	0	0	NA	0	R	0	NA	19	S	15	I
MD+20 colony 1	TH81	Pantonea	0	0	0	0	1	0	1	0	0	0	2	1	1	1	1	0	1	1	0	0	0	0	0	0	0	1	0	15	I	22	S	10	R	20	S	21	S
MD+20 colony 2	TH82	Erwinia	0	0	0	0	1	0	0	0	0	0	2	1	1	0	1	0	1	1	0	0	0	0	0	0	1	1	0	8	R	19	S	9	R	17	I	13	I
MD+20 colony 3	TH83	Bacillus	0	0	0	0	0	0	0	0	0	0	0	0	0	0	0	0	0	0	0	0	0	0	0	0	0	0	0	0	R	29	S	29	S	19	S	19	S
MD+20 colony 4	TH84	Pantonea	0	0	0	0	1	0	0	0	0	0	2	1	0	1	1	0	0	1	0	0	0	0	0	0	0	1	0	12	R	23	S	11	R	20	S	23	S
MD+20 colony 5	TH85	Bacillus	0	0	0	0	0	1	0	0	1	0	0	0	0	0	0	0	0	0	0	0	0	0	0	0	0	0	0	0	R	31	S	30	S	24	S	23	S
MD+20 colony 6	TH86	Bacillus	0	0	0	0	0	1	0	0	1	0	0	0	0	0	0	0	0	0	0	0	0	0	0	0	0	0	0	0	R	29	S	27	S	16	I	19	S
MD+20 colony 7	TH87	Bacillus	0	0	0	0	0	1	0	0	1	0	0	0	0	0	0	0	0	0	0	0	0	0	0	0	0	0	0	0	R	29	S	34	S	26	S	21	S
MD+20 colony 8	TH88	Erwinia	0	0	0	0	1	0	0	0	0	0	3	1	1	0	1	0	1	1	0	0	0	0	0	0	0	1	0	0	R	20	S	11	R	14	I	19	S
MD+20 colony 9	TH89	Bacillus	0	0	0	0	0	1	0	0	1	0	0	0	0	0	0	0	0	0	0	0	0	0	0	0	0	0	0	0	R	28	S	25	S	18	I	23	S
MD+20 colony 10	TH90	Pseudomonas	0	0	0	0	0	0	0	0	0	0	3	0	0	0	0	1	0	0	0	0	0	0	0	0	0	0	0	0	NA	30	S	25	NA	19	S	18	S
		Sum	1	1	3	1	6	20	1	4	25	40	176	8	7	2	8	35	6	7	1	2	11	1	2	2	2	6	1										

Number of copies of each gene hit in the sequence data by isolate and phenotypic results with zone of inhibition measurements (mm) and CLSI breakpoint interpretation (R-resistant; I- Intermediate; S-susceptible; NA- not applicable for due to intrinsic resistance) for Kirby-Bauer disk diffusion assay.
